# The Moderating Effect of Vaccine Hesitancy on the Relationship between the COVID-19 Vaccine Coverage Index and Vaccine Coverage

**DOI:** 10.3390/vaccines11071231

**Published:** 2023-07-12

**Authors:** Annalise Julia Tolley, Victoria C. Scott, Mary Louise Mitsdarffer, Jonathan P. Scaccia

**Affiliations:** 1 Department of Psychology, Health Psychology, University of North Carolina at Charlotte, 9201 University City Blvd, Charlotte, NC 28223, USA; 2 Department of Psychology, Health Psychology, Faculty of Psychological Science and Public Health Science, University of North Carolina at Charlotte, 9201 University City Blvd, Charlotte, NC 28223, USA; vscott10@charlotte.edu; 3 Biden School of Public Policy & Administration, Research Faculty in the Center for Community Research and Service, University of Delaware, Newark, DE 19716, USA; mmits@udel.edu; 4 Dawn Chorus Group, 342 N. Queen Street, Candy Factory Warehouse D, Lancaster, PA 17603, USA; jon@dawnchorusgroup.com

**Keywords:** vaccine hesitancy, health equity, vaccine coverage, COVID-19 Vaccine Coverage Index

## Abstract

To examine COVID-19 vaccination barriers in the US, this study drew on publicly available county-level data (n = 3130) to investigate the impact of vaccine hesitancy on the relationship between county-level social/structural barriers and vaccine coverage. A hierarchical regression was performed to establish the relationship between the COVID-19 Vaccine Coverage Index (CVAC) and vaccine coverage, assess the moderating effect of vaccine hesitancy on this relationship, and explore the influence of ethno-racial composition on vaccine coverage. A significant, negative relationship (r^2^ = 0.11, *f*^2^ = 0.12) between CVAC and vaccine coverage by county was established (step 1). When vaccine hesitancy was introduced as a moderator (step 2), the model significantly explained additional variance in vaccine coverage (r^2^ = 0.21, *f*^2^ = 0.27). Simple slopes analysis indicated a significant interaction effect, whereby the CVAC–vaccine coverage relationship was stronger in low hesitancy counties as compared with high hesitancy counties. Counties with low social/structural barriers (CVAC) but high hesitancy were projected to have 14% lower vaccine coverage. When county-level ethno-racial composition was introduced (step 3), higher proportions of white residents in a county predicted decreased vaccination rates (*p* < 0.05). Findings indicate that CVAC should be paired with vaccine hesitancy measures to better predict vaccine uptake. Moreover, counties with higher proportions of white residents led to decreases in vaccine uptake, suggesting that future intervention strategies should also target whites to reach herd immunity. We conclude that public health leaders and practitioners should address both social/structural and psychological barriers to vaccination to maximize vaccine coverage, with a particular focus on vaccine hesitancy in communities with minimal social/structural barriers.

## 1. Introduction

Each year, an estimated 2–6 million deaths worldwide are prevented by vaccines [1,2]. Vaccines reduce the incidence and severity of disease, enabling individuals to live longer, healthier lives. For instance, vaccinating just one United States (U.S.) birth cohort against 13 diseases was estimated to prevent 42,000 deaths, 20 million cases of disease, and $68.6 billion USD in societal costs [3]. This is possible because vaccines prepare one’s immune system to fight against infection, thus eradicating costly diseases, promoting herd immunity, reducing morbidity and mortality, and preventing secondary infections [1]. Given health, economic, and societal benefits, vaccine uptake should be of national interest. However, there are significant disparities in routine vaccination rates (e.g., influenza, human papillomavirus, herpes zoster, tetanus), particularly among adults. Such disparities are more pronounced along economic, racial, and ethnic lines [4,5,6,7]. For example, Hispanic/Latino, Black, Native Hawaiian/Pacific Islander, and Native American/Alaskan Native adults had lower COVID-19 vaccination rates compared with their white and Asian counterparts during the early stages of vaccine availability [8]. Such disparities are a public health and social justice concern because these minoritized populations are among the most likely to contract COVID-19, be hospitalized, and die [9,10,11].

To better understand barriers to COVID-19 vaccination that adversely affect socially vulnerable U.S. counties, and to consequently propose public health interventions to attenuate vaccination disparities, Mishra et al. [12] proposed the COVID-19 Vaccine Coverage Index (CVAC). CVAC identifies social and structural barriers to vaccine coverage across five categories: sociodemographics, resource-constrained health systems, healthcare accessibility, irregular care-seeking behavior, and historic under vaccination [13]. CVAC was then used to compare 2021 vaccine coverage in U.S. counties with high versus low barriers to immunization, as identified through the index measure [12]. Findings have indicated that high-barrier counties had vaccinated 21% fewer people than counties with the least barriers [12]. The categories and definitions of each measure that contribute to the CVAC are outlined in Table 1.

By identifying the unique barriers to vaccine coverage faced by counties, the intent of CVAC is to guide local public health professionals, practitioners, and policy makers to design targeted and efficacious interventions [13]. The underlying assumption of addressing structural barriers to vaccination elicits the *Field of Dreams* maxim, “if you build it, [they] will come.” (*Field of Dreams* is a 1989 American baseball film. In the film, the main character (Ray) hears a mysterious voice while walking through a corn field: “if you build it, he will come.” Without additional direction, Ray begins constructing a baseball field with the faith that if he constructs it, the ghosts of past baseball players will come to play). However, this may not be a reliable assumption. Though CVAC captures important county-level social and structural barriers, it does not incorporate important psychological factors such as vaccine hesitancy. Consideration of such psychological barriers as vaccine hesitancy is essential to the gaining of a nuanced understanding of what is happening at a county level. Oversight of such influences could have significant public health consequences; though, even if structural barriers are addressed and, for example, vaccines are made free and accessible, this may not be enough to promote wide-scale vaccination coverage.

Vaccine hesitancy is often defined as a behavioral phenomenon, whereby there is delayed acceptance or refusal of vaccinations despite their availability [14]. However, this behavior-focused definition omits the psychological nature of vaccine uptake. By expanding definitions to incorporate the psychological factors of hesitancy [15,16,17,18], hesitancy becomes distinct from vaccine acceptance or vaccine refusal, which are defined by behavioral outcomes. According to a systematic review conducted by Romate et al. [16], leading psychological factors associated with COVID-19 vaccine hesitancy include appraising the pandemic as lower danger, being concerned with vaccine safety, and harboring conspiracy beliefs. These COVID-19-specific psychological factors are consistent with vaccine hesitancy more broadly, which is influenced by misinformation [19,20,21,22], political ideology [23,24,25,26], and medical mistrust of providers, healthcare systems, and pharmaceutical companies [27,28]. This array of psychological barriers may be conceptualized as the “3Cs” proposed by the World Health Organization [14]: complacency (i.e., perceived risk and cost–benefit analysis of vaccination), convenience (i.e., perceived quality of the service and appropriateness within cultural context), and confidence (i.e., trust in the vaccine, health system, public health officials). To reflect the psychological essence of vaccine hesitancy, this paper defines vaccine hesitancy in terms of the psychological factors weighed by an individual that might lead to a reluctance to accept a vaccine, despite it being readily available.

While psychological factors for vaccine hesitancy differ based on sociodemographic characteristics, time, and context [29], the ultimate impact of vaccine hesitancy is the same: reduced vaccine uptake and diminished herd immunity [30,31,32,33]. Thus, when making decisions about public health policy and interventions that aim to promote vaccine uptake at the county-level, it is essential to consider social and structural barriers to vaccination as well as psychological barriers. However, no known studies have investigated the influence that vaccine hesitancy has on county-level vaccine coverage in light of social and structural barriers. If vaccine hesitancy moderates the relationship between structural barriers and vaccine coverage at the county level, then merely addressing structural barriers to vaccine access may be insufficient to promote large-scale vaccine coverage.

### Study Aims

This study is guided by two research questions: (1) do county-level social and structural barriers, as measured by CVAC, predict county level vaccination rates? (2) Does vaccine hesitancy moderate the relationship between social and structural barriers and vaccine coverage at the county level?

## 2. Materials and Methods

### 2.1. Data

This study uses publicly available county-level, cross-sectional data from the U.S. Department of Health and Human Services Household Pulse Survey (HPS) and the Centers for Disease Control and Prevention’s (CDC) county-level COVID-19 Vaccinations in the United States. HPS data contain predictions of vaccine hesitancy rates, CVAC scores, and racial and ethnic composition, all at the county-level [34]. These data were merged (using FIPS county code) with COVID-19 vaccination data containing the percent of fully vaccinated adults in a given county as of 13 April 2022 [35].

### 2.2. Measures

For analyses, we examined county-level vaccination, CVAC, vaccine hesitancy, and county-level racial and ethnic characteristics. The main outcome of interest was county-level vaccine coverage. Vaccination coverage was defined in accordance with CDC standards as the percentage of adults in a county who were fully vaccinated as of 13 April 2022 (the proportion of those who obtained the second dose of a two-dose vaccine or one dose of a single-dose vaccine [35]).

Indicator variables included CVAC and vaccine hesitancy. CVAC is a modular index with a number of indicators that reflect five thematic barriers to vaccine coverage: (i) historic undervaccination, (ii) sociodemographic barriers, (iii) resource-constrained health systems, (iv) healthcare accessibility barriers, (v) irregular care-seeking behavior [13]. Indicator values were transformed into percentiles so that the final, aggregate score can be presented on a continuous, 0 to 1 scale (0 = least concerning, 1 = most concerning) [13]. Additional information on the CVAC methodology, as designed by Surgo Ventures, is publicly available [13].

The CDC estimated COVID-19 vaccine hesitancy using the HPS survey question, “once a vaccine to prevent COVID-19 is available to you, would you… get a vaccine?” [36]. These data were collected before the vaccine was available. Responses were indicated on a five-item Likert scale from “definitely get a vaccine” to “definitely not get a vaccine” [36]. Answers were coded into categories: with those responding “probably not,” “definitely not,” or “unsure” fitting into the hesitant/unsure category [36]. County-level hesitancy rates were defined by the percentage of the population that endorsed the hesitant/unsure category. These data were estimated in a multi-step process documented by the CDC and using the PUMA-to-County crosswalk established by the Missouri Census Data Center [36].

County-level racial and ethnic demographic data were obtained from the 2019 American Community Survey 5-year estimates and included the following categories: Hispanic, non-Hispanic white, non-Hispanic Black, non-Hispanic American Indian/Alaska Native, non-Hispanic Asian, and non-Hispanic Native Hawaiian/Pacific Islander [34].

### 2.3. Data Analysis Plan

For inclusion in the sample, counties must have had county-level data for the variables of interest: vaccine coverage, CVAC, vaccine hesitancy, and ethno-racial composition (n = 3130). Any counties with incomplete data were excluded from our sample (n = 12).

R statistical software (version 3.6.3) was used to test assumptions for descriptive and inferential data analyses (packages: lsr, psych, car, stats, interactions, ggplot2, dplyr). Before conducting the linear regression, assumptions were tested. Cook’s distance and the leverage of studentized residuals determined outliers in CDC county-level COVID-19 vaccination data. Identified outliers were investigated for accuracy by Author 1 by referring to another set of local- or state-reported data. In instances where the CDC’s data were inaccurate, county-level data were used (n = 7) [37,38]. Counties with vaccination rates greater than 90% (n = 11) were also compared with an additional source of local or state data to ensure accuracy. Two counties were identified as having data entry mistakes (i.e., instead of reporting adults fully vaccinated, the total number of doses were reported, thus artificially inflating the percentage) [39,40]. Additional outlier testing identified six counties as potential outliers, but none had significant influence based on Cook’s distance. Analytical analyses were run with and without these counties as a robustness check: outliers did not substantially change the model when included. Therefore, outliers were included in the final analysis because the data are meant to be nationally representative of the U.S. population. Assumption checks indicated normality, linearity between predictors and outcomes, and homoscedasticity of the residuals. Multicollinearity was not deemed an issue.

Hypotheses were tested using hierarchal, linear regression modeling with the indicator variables as CVAC and vaccine hesitancy and vaccine coverage as the outcome variable. A bi-variate regression between CVAC and county-level vaccination was conducted (Step 1). Then, vaccine hesitancy was introduced into a multiple regression to determine moderation (Step 2). Finally, county-level race and ethnicity data were entered into the multiple regression to control for the influence that ethno-racial composition in a given county may have on vaccination rate (Step 3). Additionally, a simple slopes analysis was run to visually assess the interaction effect between the CVAC and vaccine hesitancy and its influence on county-level vaccination rates (Step 2). Effect sizes were evaluated using Cohen’s f^2^ [41]. Partial effects were evaluated in accordance with Hair, Jr. and colleagues [42].

## 3. Results

Table 2 provides descriptive characteristics of the counties and district (Washington, DC, USA) within the sample (n = 3130). The average vaccination rate by county was 50.8%, ranging from 11.1 to 95.0% of the total population. Across counties, 19.2% of the population was, on average, considered vaccine hesitant. CVAC scores ranging from the minimum possible (0) to the maximum possible (1) are also reflected in the sample, with an average CVAC score of 0.5 (SD 0.29). On average, counties had a white population consistent with the national rate of about 76%. However, minoritized racial and ethnic group composition varied to a considerable degree depending upon the county. For example, in some counties, there was no proportion of the population identifying as Hispanic/Latino, while other counties, such as Starr County, Texas, reflected a population that was 99.2% Hispanic/Latino.

Table 3 shows the results of our hierarchal, multiple regression. In step 1, we introduced a bivariate regression between CVAC and vaccination rates, and our findings indicate that, as CVAC increased, there was a significant, negative decrease in county-level vaccination rates (*b =* −0.14, *p* < 0.001). The observed effect was small (*f*^2^ = 0.12). Step 2 introduced vaccine hesitancy and the interaction term between CVAC and vaccine hesitancy to our model. Once again, we found that, as CVAC increased, there was a significant, negative decrease in vaccination rates at the county-level (*b =* −0.26, *p* < 0.001). However, with the inclusion of vaccine hesitancy in the model, the observed effect was medium to large (*f*^2^ = 0.27). When the percentage of a county expressing vaccine hesitancy increased, there was an even greater, significant decrease in county-level vaccination rates (*b =* −1.14, *p* < 0.001) Finally, step 3 included county-level, demographic data regarding the percentage composition of racial and ethnic groups. Ceteris paribus, one-unit increases in CVAC scores (*b =* −0.28, *p* < 0.001) and vaccine hesitancy (*b =* −0.65, *p* < 0.001) remained significantly associated with decreases in vaccination rates at a county level. The observed effect was extremely large (*f*^2^ = 0.64). When looking at county-level demographic compositions, and controlling for all other factors, an increase in the percentage of the Asian population (*b =* 0.77, *p* < 0.001) of a given county was significantly associated with increased vaccination rates. Conversely, when holding all other factors constant, an increase in the percentage of white (*b =* −0.27, *p* < 0.05) and Native Hawaiian/Pacific Islander (*b =* −3.22, *p* < 0.001) of a given county were significantly associated with decreased vaccination rates. Finally, our analysis did not find that the percentage of American Indian/Alaska Native, Black, or Latino residents at a county-level were significant when predicting county vaccination rates when all other variables were held constant.

**Figure 1:**
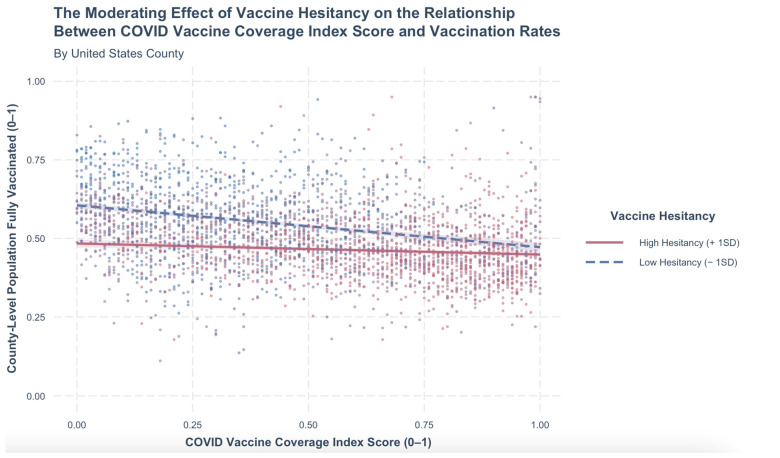
The moderating effect of vaccine hesitancy on the relationship between the COVID-19 Vaccine Coverage Index (CVAC) and vaccine coverage by county. Whereby a CVAC score of zero indicates no social/structural barriers to vaccination and one indicates the most social/structural barriers to vaccination at a county level.

Figure 1 illustrates the relationship between vaccine hesitancy and CVAC county-level vaccination (Table 3, step 2), the regression coefficient of the interaction term was statistically significant from step 2 of the hierarchal regression model (*b* = 0.92, *p* < 0.001). The size of this interaction effect is very large for moderation analysis (*f*^2^ *=* 0.13; [42,43]). To determine the nature of the interaction, as it contains two continuous variables, a simple slopes analysis was conducted. In support of the hypothesis, at lower levels of vaccine hesitancy, the relationship between CVAC and vaccination rates was greater (*b* = −0.13, *p* < 0.001) than at higher levels of vaccine hesitancy (*b* = −0.04, *p* < 0.001). High and low vaccine hesitancy was determined by one standard deviation above and below the mean. As is illustrated in Figure 1, high vaccine hesitancy attenuates the relationship between CVAC and the percentage of the vaccinated population in U.S. counties in our sample. This model predicts that a county with high vaccine hesitancy and no social and structural barriers has a vaccination rate up to 14% lower than a county with low vaccine hesitancy and no social and structural barriers.

## 4. Discussion

In the words of Cutler and Summers [44], the COVID-19 pandemic “is [among] the greatest threat to prosperity and wellbeing facing the United States since the Great Depression.” The estimated financial toll to the U.S. is well above 10 trillion dollars, which includes the cost of gross domestic product loss, educational disruption, chronic health conditions, mental health loss, and lives lost [45]. In addition to the economic burden of lives lost, there is an emotional toll incurred. This is especially true for socially vulnerable groups, who—in the absence of health-promoting social and structural determinants of health—disproportionately represent higher rates of mortality [46].

This study illuminates three key insights about vaccination coverage among U.S. counties. First, while CVAC does modestly predict vaccine coverage by county, the model is strengthened significantly when vaccine hesitancy is included. This indicates that CVAC alone, a measure of county-level social and structural barriers to vaccination coverage, paints only part of the vaccine coverage picture. Therefore, public health leaders and practitioners would be remiss to focus vaccination coverage interventions solely on social and structural barriers and should instead consider the interplay between social and structural barriers and psychological barriers, reflected by the addition of vaccine hesitancy. Additional research on the utility of the CVAC is essential, as it was designed as a practical tool for practitioners and policy makers; however, only eight publications mentioning CVAC currently exist as of June 2023, and none (beyond the article written by its creators) discuss its practical utility.

Second, results indicate that vaccine hesitancy significantly moderates the relationship between CVAC and vaccine coverage, and that the observed effect is large. Under conditions of low vaccine hesitancy, counties with minimal social and structural barriers have a predicted vaccination rate above 62%. However, under conditions of high vaccine hesitancy, counties with minimal social and structural barriers have a predicted vaccination rate below 50%. This finding has two key implications. One implication is that simply removing social and structural barriers to vaccination may only lead to a modest increase in vaccine coverage. The other implication is that socially and structurally advantaged counties that are hesitant about vaccines hinder the overall vaccine coverage nationwide. Therefore, it is crucial to address the barriers identified by CVAC and tackle vaccine hesitancy through targeted science communication and other interventions. This can be achieved by harnessing the power of the media, partnering with trusted community-based organizations, and increasing the visibility of vaccination within social groups. By doing so, we can effectively promote population health and achieve herd immunity, especially in counties with minimal social and structural barriers to vaccination.

Third, results indicate that, when the model includes county-level differences in race and ethnicity, as vaccine hesitancy and CVAC are held constant, counties with larger proportions of white people are predicted to be less vaccinated than counties with lower proportions of white people. This finding is mirrored in the Native Hawaiian/Pacific Islander population, whereby an increase in the proportion of a county’s Native Hawaiian/Pacific Islander population predicts a decrease in vaccination. However, the implications for these two populations are different. On average, Native Hawaiian/Pacific Islanders account for 1% of county populations, whereas whites account for an average of 76.3% of the county populations. Therefore, whites have a bigger impact on population health due to their plurality, and low vaccination rates in this group may be meaningful to the spread and persistence of COVID-19. Therefore, to increase nationwide vaccine uptake, intervention strategies should begin to target white populations who are reluctant to vaccination despite availability.

These findings—lower vaccine uptake among counties with a large proportion of white individuals—are in contrast to studies using 2021 data, which found that lower vaccination coverage was present among Black and Latino communities [47,48,49,50]. However, this study uses April 2022 data, and results are consistent with research by Wu [50], which indicate that, over time (2021 to 2022), the association between white concentration and vaccination went from highly positive to highly negative. These results help to explain why population-level disparities in U.S. vaccine coverage diminished between racial and ethnic groups by the end of 2021, once the vaccine had become more widely available [8]. Likely, as interventions addressing social and structural barriers to vaccination within minoritized ethno-racial groups were implemented, vaccine uptake increased in these groups. Conversely, many white communities may have had greater access to vaccination due to its early rollout as a result of structural racism and socioeconomic advantage [51,52,53,54]. Thus, lower vaccine uptake overtime must be attributable to psychological factors, which, according to our results and corroborated by Hu et al. [55], cannot be fully explained by vaccine hesitancy (when using HPS data).

To understand which psychological factors may be influencing the choice of predominantly white communities to not get vaccinated, we surveyed the literature. While there is a plethora of studies that focus on the barriers to vaccination faced by minoritized racial and ethnic groups [56,57,58,59,60], there is a dearth of research investigating psychological barriers to vaccine uptake among white Americans. Newer studies suggest that political ideology, rugged individualism, mediate intake and moral concerns may be factors to explore in additional research [50,61,62,63]. This gap in our knowledge has detrimental implications for population health in the U.S. If we do not understand how to increase vaccine uptake among the racial group that is the numerical majority in the U.S., it will continue to be difficult to promote herd immunity. Thus, the public health consequences of uncontrolled disease will disproportionately burden marginalized populations [9,10,11].

### 4.1. Implications for Health Equity

As a matter of health equity, barriers to vaccination are important to address because they disproportionately hinder marginalized populations from vaccination, who also tend to be at the greatest risk for bearing the disease burden. However, as a matter of public health, the goal of vaccination is to reach herd immunity, which also requires vaccination among structurally and socially advantaged groups. Herd immunity is a path to achieve health equity. By reducing the spread of the virus, the entire population benefits, but especially those who face the greatest risk of morbidity and mortality. In the words of Williams and Cooper [64], “COVID-19 disparities are not the fault of those who are experiencing them, but rather reflect social policies and systems that create health disparities in good times and inflate them in a crisis”.

The vast majority of studies and interventions seek to understand lower vaccination rates among marginalized groups [56,57,58,59,60,65], but the results of this study importantly tell the part of the story that is often missed in health equity research: vaccination responsibility cannot be placed entirely on the shoulders of those experiencing health inequities, and socially vulnerable populations are not the only demographic group in which to invest research and intervention resources. Populations with social and structural advantages (white, high income, access to routine healthcare, health insurance coverage, etc.) also bear vaccination responsibility and are key segments of the population to target in order to promote population health.

### 4.2. Implications for Practice

The generalizable take-away from the lackluster COVID-19 vaccine uptake, is that removing social and structural barriers is not enough to reach herd immunity and promote population health in the U.S. Psychological barriers, which are reflected in measures of vaccine hesitancy, must also be addressed. However, this can be challenging in practice due to the various psychological factors behind vaccine hesitancy that are weighted differently by individuals.

To characterize the major sources of vaccine hesitancy and to propose tailored solutions, Surgo Ventures [66] identified five vaccine personas that may shed light on targeted interventions to increase vaccine coverage: the enthusiasts, the watchful, the cost-anxious, the system distrusters, and the skeptics. Their proposed personas include both psychological and social and structural barriers. For example, the enthusiasts have one key barrier: availability [66]. For this group, if you build it, they will come. On the other hand, system distrusters are worried about vaccine safety and distrust medical systems [66]. For this group, targeted intervention may include partnering with trusted community-based organizations to provide education related to the safety of the vaccine (e.g., what is a vaccine, why is it safe despite the quick development, what does it mean for the FDA to approve a vaccine). Unlike system distrusters, the watchful—individuals who make their vaccination decisions based on community norms—will require a solution that underscores vaccination as a positive social norm [66]. This tool demonstrates the multitude of social, structural, and psychological barriers that must be addressed by future public health interventions to increase vaccine uptake.

Use of these vaccine hesitancy personas has shown promise for successful intervention. Surgo Ventures and Mary’s Center [67] partnered to develop a vaccine ambassadors toolkit that helped 200 additional people get vaccinated during a two-month pilot. They used these personas to craft targeted messages that vaccine ambassadors (i.e., trusted messengers) could share with their social networks based on the root of their hesitancy [67]. For example, system distrusters were addressed with information that emphasized the safety of the vaccine and the research that points to its efficacy. Conversely, messaging to the watchful emphasized the popularity of the vaccine to bolster one’s perception of vaccination as a social norm [67]. A toolkit such as this could serve as a useful framework regardless of the vaccine. County-level infrastructure to identify these five personas and roll out interventions is likely to be salient for a faster response when the next pandemic inevitably strikes.

### 4.3. Limitations

A number of limitations are important to note along with the study findings. Vaccine hesitancy data were collected at a different time point than the vaccination data. Differences in timing reflect a lack of updated, nationally representative hesitancy data after the COVID-19 vaccine became available in late 2020. Therefore, the assumption is that individuals who reported being vaccine hesitant before availability remained vaccine hesitant after the vaccine had become available. Consequently, the regression models should be interpreted with caution. While this is a disadvantage, the authors determined that it was valuable to include nationally representative data (of which no more recent vaccine hesitancy data exists). Another limitation is that the CDC estimated county-level vaccination rates using 2010 Census data, instead of American Community Survey (ACS) data. Census data reflect the most accurate count of individuals in a given county every 10 years, whereas ACS data are collected yearly and reflect estimates from randomized county samples. Because the U.S. population has grown since 2010 [68], each county-level vaccination rate may be an overestimate or underestimate of the current population [69]. Additionally, there may be biases in the reporting of county-level aggregate data that should be considered when evaluating the study results. A third limitation is that our analyses utilized cross-sectional data, which reflect the population at a given point in time. As such, we are only able to report the relationship between vaccine hesitancy and our indicator variables, and not causal pathways. Lastly, there are a number of indicators that influence vaccine hesitancy and vaccination, many of which are encompassed by the CVAC index (e.g., education, income, socially vulnerability, etc.). However, this study did not isolate these additional indicators or control for them in the regression model.

## 5. Conclusions

While the release of the COVID-19 vaccine was heralded as a scientific success in vaccine production, its public health value was dampened by modest national uptake as compared with other countries [70,71]. Some studies suggest that 80–90% of the population must be fully vaccinated to achieve herd immunity against COVID-19 [72,73], but at the time the data were collected, only 66% of the population was fully vaccinated [74].

As of May 2023, over one million lives in the U.S. have been lost due to the pandemic, with more than 50% of those losses occurring after the availability of the vaccine [75]. A disproportionate number of those lost identified as Hispanic/Latino, Black, Native Hawaiian/Pacific Islander, and American Indian/Alaskan Native populations [76,77]. While social and structural inequities are major factors in these disparities [46], this study underscores that addressing social and structural barriers to vaccination are not enough to promote population health when psychological factors such as vaccine hesitancy persist. Therefore, to see the greatest public health impact, we must also design vaccination interventions that address vaccine hesitancy, especially among socially and economically advantaged populations—who have a major impact on population health—such as counties with minimal social and structural barriers or large proportions of white people. Otherwise, viral surges will continue to have the greatest negative impact on socioeconomically disadvantaged groups [11,78]. Future research should continue to identify the major sources of vaccine hesitancy within each county and develop targeted interventions when the next pandemic inevitably hits, not only among the most vulnerable groups, but also less vulnerable groups who, nevertheless, greatly influence population health.

## Figures and Tables

**Table 1:** Indicators for the COVID-19 Vaccine Coverage Index.

Barriers *	Definition
Historic Undervaccination	Lower coverage and higher refusal rates based on the proportion of children and adults that have routine vaccinations, such as MMR, polio, HPV, flu
Sociodemographic Barriers	Proportion of the population socio-economically disadvantaged based on census data such as unemployment, household income, education, proportion of racial and ethnic minority groups
Resource-Constrained Health System	Healthcare system has less available healthcare staff and funding, lower quality of care, and less infrastructure of vaccine administration per capita
Healthcare Accessibility Barriers	Proportion of population with barriers to healthcare such as cost and transportation
Irregular Care-Seeking Behavior	Proportion of population without routine access to providers and/or healthcare systems

* Indicators/barriers according to Surgo Ventures development of the CVAC [13].

**Table 2:** Aggregate county-level descriptive statistics.

	Mean (SD)	Min.	Max.
CVAC Score	0.5 (0.29)	0.0	1.0
% Vaccinated (April 2022) *	50.8 (11.9)	11.1	95.0
% Vaccine Hesitant *	19.2 (5.3)	5.0	32.3
Racial/Ethnic Demographics *			
% Hispanic/Latino	9.4 (13.9)	0.0	99.2
% American Indian/Alaskan Native	1.8 (7.6)	0.0	91.9
% Asian	1.4 (2.7)	0.0	41.7
% Black	9.0 (14.4)	0.0	87.2
% Native Hawaiian/Pacific Islander	1.0 (0.4)	0.0	11.1
% White	76.3 (20.2)	7.0	100.0
N = 3130			

* While all of the variables in this study are on a 0–1 scale, the data have been converted to percentages for Table 2.

**Table 3:** Relationship between CVAC, vaccine hesitancy, racial/ethnic demographics, and vaccine coverage by county.

	(1)	(2)	(3)
CVAC	−0.14 ***	−0.26 ***	−0.28 ***
	(0.01)	(0.03)	(0.03)
Vaccine Hesitant		−1.14 ***	−0.65 ***
		(0.07)	(0.07)
CVAC: Hesitant		0.92 ***	0.50 ***
		(0.13)	(0.13)
Racial/Ethnic Demographics			
Hispanic/Latino			−0.01
			(0.12)
American Indian/Alaska Native			0.09
			(0.12)
Asian			0.77 ***
			(0.15)
Black			−0.10
			(0.12)
Native Hawaiian/Pacific Islander			−3.22 ***
			(0.55)
White			−0.27 *
			(0.12)
Constant	0.58 ***	0.76 ***	0.93 ***
	(0.00)	(0.01)	(0.11)
F-Statistic	397.2 ***	269.5 ***	220.4 ***
Adjusted R-Squared	0.11	0.21	0.39

* Standard errors in parentheses. Statistical significance indicated as *** *p* < 0.0001, * *p* < 0.05. Observations are the same across all regressions (n = 3130). Values listed are unstandardized regression coefficients (*b*).

## Data Availability

Publicly available datasets were analyzed in this study. These data can be found online at https://data.cdc.gov/Vaccinations/COVID-19-County-Hesitancy/c4bi-8ytd (accessed on 15 April 2022) and at https://data.cdc.gov/Vaccinations/COVID-19-Vaccinations-in-the-United-States-County/8xkx-amqh (accessed on 15 April 2022).
